# Pulp Capping

**Published:** 1877-04

**Authors:** W. C. Barrett


### ARTICLE VI.

### *Pulp Capping.*

[The following letter, on the vexed question of Pulp Capping, written by Dr. W. C. Barrett, in reply to that of a brother dentist, will be read with interest. It will be seen that the writer takes issue with the present usual practice of treating exposed pulp tissue, and states his reasons therefor in a forcible manner.—EDITOR.]

MY DEAR DOCTOR:—Your letter, containing a beautiful specimen of recalcification of dentine, under the protecting influences of an oxychloride of zinc filling, is at hand. The case illustrates what function can do. But for one such instance of a return to a true pathological condition, I fear the ninety and nine are but sleeping volcanoes, which may at some time, after horrible intestinal disturbances, burst and bring destruction in the train of sequences. Bitter experience has taught me that living tissue cannot tolerate chloride of zinc. It is an exasperating irritant. Years of faithful trial and careful observation, have afforded abundant reasons for changing a former opinion, too hopefully entertained and incautiously expressed. Bitterly have I repented my credulity. Let me here say, however, that I have not abandoned the use of this material, though I no longer permit chloride of zinc to come into the immediate vicinity of exposed pulp tissue. I frequently succeed in saving an aching tooth alive, and when it is accomplished, I thank God and take courage. But in such cases, I triumph *not* by introducing irritants into an already disturbed territory—not by fanning an existing flame, but by precisely the opposite method. It is simply incredible, how we have persisted in the attempt to stop a conflagration, (inflammation: *in-flammare*, to flame) by stirring up the fire. It is high time that we traveled in some other direction, for in the main, we are all far from the goal of our ambition. It is only such an occasional success as this which you chronicle, that has encouraged us to persevere in our thorny path.

Either you or I are very much mistaken, in the view we take of the therapeutical action of creasote and carbolic acid. You recommend their application to the exposed pulp tissue, that they may, as you say, unite with it, and thus form an insoluble pellicle (carbolate of albumen), which will protect the pulp. Protect it from what? From the oxy-chloride of zinc? If that is the object, you admit the point at issue; which is the question whether that substance is an effective shield. If the pulp must be protected from its protector, pray where is the security? And just here is my misgiving. Have we all this time been endeavoring to save pulps, by turning them over to the care of their worst enemy? Is it the old fable of the Kite, the Hawk, and the Doves, over again? You remember—"*At in columbare receptus, uno die, majorem stragem quam milvius longo tempore.*" Shall we never heed the good old "*Haec fabula docet*," of our school boy days? If then, the dental Dove needs to be protected against the chloride Kite, are we sure that we have not brought in a carpolate Hawk to do it? Creasote is a cauterant, and destroys pulp tissue. It burns it and leaves an eschar as the result, which does not materially differ from that left by grangrene or sphacelus. This cauterizing creates, even in healthy tissue, an inflammation which results in pus between the living and dead, and this in turn causes a slough of the cauterized crust or scab. Yet you would after bringing about this state of affairs, securely confine this slough against elimination, by imprisoning it beneath walls of oxy chloride of zinc.

Let us see how reasonable appears your theory. There is, in the tooth in question, a violent state of inflammation. To sooth this, you apply one of the most violent irritants you are acquainted with—so violent in its action that it entirely destroys a part of the life you are attempting to save. Lest, however, this be not sufficient, you introduce immediately upon this sore spot, over the destroyed and unsloughed tissue, another violent irritant, that the work left incomplete by the first agent, may be finished by the second. Pray, what kind of a way is this to soothe to quiet rest an exacerbated member? Were I in a state of mental irritation, I should not like to have you approach me with such psychological emollients? I scarcely can conceive of a course that would seem more certain to result in loss of a tooth, than such an one. Think of it—in an endeavor to make sensitive tissues tolerate an unendurable substance, you destroy the vitality of that with which it must come in contact, and leave offensive, putrefying corpse for the peace-maker. Is this the pacifying, tranquilizing process, which shall bring peace to these quarreling elements? Pray! *why* do dentists apply creasote in cases of simple inflammation? *What* therapeutical action has it that it should be indicated? If there be putrescence, or sloughing, then I grant you its exhibition; but for simple inflammation, or congestion of the pulp—NEVER.

Let us get more into line with physiological action. It is useless to fight against the unceasing activities of nature. We must place ourselves parallel with her path, and not get directly athwart her course, and endeavor to force her into our worn ruts. If a pulp is to be capped, the alarmed and irritated tissue must be first lulled to sleep, and then the operation so delicately performed, as not to waken it. If it once finds out that it has any other than its natural bed-cover, farewell to any hopes of permanent peace. The amount of it is, the whole system of pulp capping is unnatural, because it is an attempt to establish abnormal relations. Like trepaning, it is a successful effort to perform an impossibility. Would I then abandon all hope of saving exposed pulps? By no means. While trepaning is successfully performed, pulp capping may be. But the former is not done by cauterizing the brain, and then immediately placing an iraitant in immediate contact, and immovably confining it—nor can the latter be successfully done in the same manner, in any other than exceptional cases. The agent for properly capping exposed pulps, has not, I think, as yet been introduced to the profession. It will be discovered, for many eyes are turned in that direction ; but when found, it will not be an exasperating irritant. The latest aspirant to the honor is Lacto Phosphate of Lime. With this I have experimented considerably. I am not prepared to either deny, or assert that there may be a crystallization of the lime among the fibres of decalcified dentine—a process tantamount to a recalcification. I think I have seen indications of such a process. But that absorbtion, assimilation, and reproduction, may all be carried on in an outlying dependency, I cannot for a moment believe. There may, however be some kind of a calcifying process that will answer the same ends. *Calcis Phos.* is certainly a more congenial material than *Oxy-Chl. Zinc*, and it is worth while to try it. But I have no faith in *specifically* applying anything to an inflamed pulp for a covering. It must first be restored to a comparatively healthy condition by soothing applications—the use of *tinct aconite rad.*, or other anti-phlogistic remedies. Then comes the opportunity to attempt the bridging of the chasm by capping. If in the course of this process the sleeping lion be again aroused, there is nothing to do but to commence *de novo.* We shall sometimes fail through ignorance or awkwardness, but there is nothing for us to do but to keep trying, till the long looked for desideratum is gained, and we can cap and permanently fill over an exposed pulp, with some assurance of permanent success.

Most truly yours, W. C. Barrett.

—*Dental Advertiser.*

---

